# Proton Irradiation Effects on the Time-Dependent Dielectric Breakdown Characteristics of Normally-Off AlGaN/GaN Gate-Recessed Metal-Insulator-Semiconductor Heterostructure Field Effect Transistors

**DOI:** 10.3390/mi10110723

**Published:** 2019-10-26

**Authors:** Dongmin Keum, Hyungtak Kim

**Affiliations:** School of Electronic and Electrical Engineering, Hongik University, Seoul 04066, Korea; rmaehdalf@hanmail.net

**Keywords:** AlGaN/GaN, proton irradiation, time-dependent dielectric breakdown (TDDB), reliability, normally off

## Abstract

In this work, we investigated the time-dependent dielectric breakdown (TDDB) characteristics of normally-off AlGaN/GaN gate-recessed metal–insulator–semiconductor (MIS) heterostructure field effect transistors (HFETs) submitted to proton irradiation. TDDB characteristics of normally-off AlGaN/GaN gate-recessed MISHFETs exhibited a gate voltage (*V*_GS_) dependence as expected and showed negligible degradation even after proton irradiation. However, a capture emission time (CET) map and cathodoluminescence (CL) measurements revealed that the MIS structure was degraded with increasing trap states. A technology computer aided design (TCAD) simulation indicated the decrease of the vertical field beneath the gate due to the increase of the trap concentration. Negligible degradation of TDDB can be attributed to this mitigation of the vertical field by proton irradiation.

## 1. Introduction

Semiconductor technology used in satellites or exploration robots in harsh environments is mainly based on silicon semiconductor technology, but uses modules for heat dissipation, the hermetic structure, and the shielding structure for extreme environments, such as high temperature and radiation; however, these modules are generally heavy and complicated parts. This burden can be relieved if robust semiconductor materials can be employed in the electronics used for harsh environments. Among attractive candidates, AlGaN/GaN heterostructure field effect transistors (HFETs) are attracting much intention as power switching devices for harsh environmental applications thanks to GaN’s superior radiation resistance [1]. Recently, studies on the radiation characteristics of GaN-based transistors have been widely conducted [2,3,4]. Especially, studies on the irradiation effects of protons occupying the majority of low earth orbits (LEO) were carried out [5,6,7]. In general, AlGaN/GaN HFETs irradiated with protons exhibit a positive shift in the threshold voltage (*V*_th_) and a reduction in the drain current (*I*_DS_), which can be attributed to the displacement damage near the two-dimensional electron gas (2-DEG) [8,9]. Exceptionally, the improvement of carrier concentration also has been reported at a relatively low dose [10]. In this paper, a sufficiently high dose (5 × 10^14^ cm^−2^) was used to deteriorate the irradiated devices and the irradiated devices followed the generally reported results [11].

The main advantage of an AlGaN/GaN heterostructure is a natural formation of a 2-DEG channel without intentional doping [12], which leads to high mobility with a high sheet charge density [13]. Therefore, AlGaN/GaN HFETs inherently operate as normally-on devices. However, for the circuit configuration and stable operation, it is essential to implement a normally-off operation [14]. A gate-recessed metal–insulator–semiconductor (MIS) structure was employed to realize a normally-off operation and has exhibited a stable *V*_th_ over 1 V and a low gate leakage [15,16]. Gate reliability has been one of the critical issues of AlGaN/GaN HFETs and can be aggravated in gate-recessed MIS structures due to the processes of AlGaN barrier etching and insulator deposition [17,18,19]. In this study, we fabricated normally-off AlGaN/GaN MISHFETs by using a gate-recessed MIS structure and investigated the effects of proton irradiation on the gate reliability of normally-off AlGaN/GaN gate-recessed MISHFETs.

## 2. Experimental

**Figure 1:**
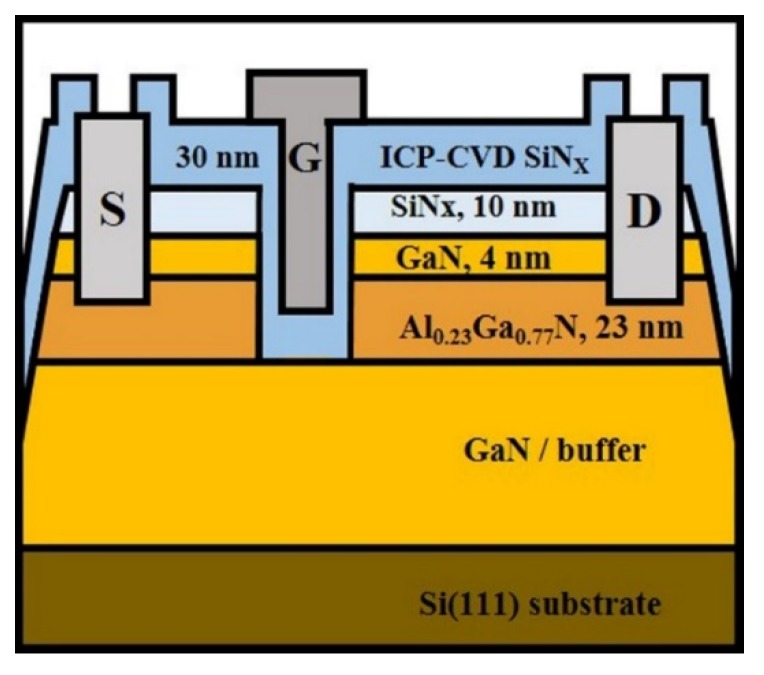
The cross-sectional view of the fabricated device. (S = Source, G = Gate, D = Drain, ICP-CVD = Inductively coupled plasma-chemical vapor deposition).

AlGaN/GaN-on-Si wafer was provided by Enkris, Suzhou, China. As presented in the data sheet from Enkris, the epitaxial structure, grown using metal organic chemical vapor deposition (MOCVD) (AIXTRON, Herzogenrath, Germany), consisted of a 10-nm in situ SiN_x_ layer, a 4-nm undoped GaN capping layer, a 23-nm undoped Al_0.23_Ga_0.77_N barrier, and 5-μm undoped-GaN buffer layer on a GaN-on-Si substrate. A cross-sectional diagram of the fabricated MISHFET is shown in Figure 1.

The fabrication process was as follows. Ohmic contacts were formed using an e-beam evaporated Ti/Al/Ni/Au (20/120/25/50 nm) metal stack and alloyed via rapid thermal annealing at 830 °C for 30 s. After the ohmic process, the mesa isolation and gate recess followed. The AlGaN barrier was fully recessed using inductively coupled plasma–reactive ion etching (ICP-RIE) (BMR Technology Corporation, Placentia, CA, USA) with a power of 5 W and a Cl_2_/BCl_3_ ambient atmosphere. A 30-nm SiN_X_ layer was deposited using ICP chemical vapor deposition (ICP-CVD) ( BMR Technology Corporation, Placentia, CA, USA) using SiH_4_/NH_3_ gas at 350 °C, and a Ni/Au (40/350 nm) metal stack was evaporated for the gate contact. The gate length/width, gate-to-drain distance, and gate-to-source distance were 2/100 μm, 15 μm, and 3 μm, respectively.

TDDB characteristics were measured using the gate voltages of 13, 13.5, and 14 V at the temperature of 150 °C. Proton irradiation was carried out using a MC-50 cyclotron (Scanditronix, Vislanda, Sweden) at the Korea Institute of Radiological and Medical Sciences (KIRAMS) with an energy of 5 MeV, and a total fluence of 5 × 10^14^ cm^−2^ was chosen to deteriorate the irradiated devices. Proton irradiation was performed at room temperature. Electrical characteristics and cathodoluminescence (CL) were measured using a Agilent 4155A semiconductor parameter analyzer (Agilent Technologies, Santa Clara, CA, USA) and a JXA-8530F (JEOL Ltd., Tokyo, Japan), respectively.

## 3. Results and Discussion

**Figure 2:**
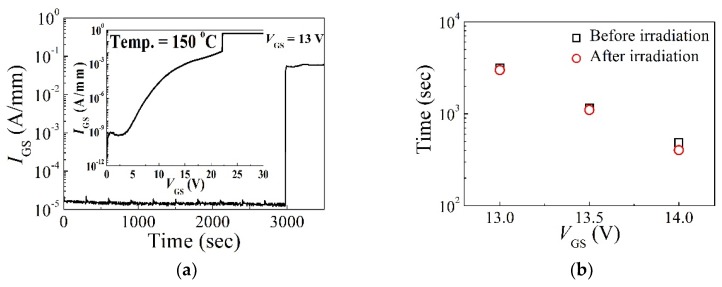
(**a**) The results of time-dependent dielectric breakdown (TDDB) characteristics carried out on one representative device (inset) time-zero breakdown (TZB) characteristics. (**b**) The *V*_GS_ dependence of TDDB characteristics at 150 °C with *V*_GS_ = 13, 13.5, and 14 V.

Figure 2a shows a representative result of the time-zero breakdown (TZB) and TDDB characteristics with *V*_GS_ = 13 V at 150 °C. For TDDB measurements, we used the constant voltage method. In the constant voltage method, the gate voltages close to TZB were applied and the gate current (*I*_GS_) was measured periodically. The gate current typically decreased before the time-dependent breakdown. *I*_GS_ increased after certain period of time and this time was defined as the time to breakdown (*t*_BD_) [20]. In order to investigate the TDDB characteristics of normally-off AlGaN/GaN gate-recessed MISHFETs, we carried out constant voltage stress tests with gate voltages of 13, 13.5, and 14 V. Figure 2b shows the relationship between the TDDB and *V*_GS_ before and after the proton irradiation. TDDB characteristics showed an almost negligible change, although the devices were irradiated with protons.

**Figure 3:**
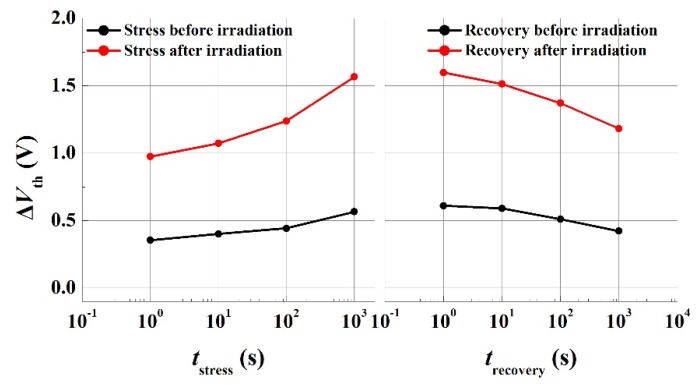
Stress and recovery behavior of normally-off AlGaN/GaN gate-recessed metal–insulator–semiconductor heterostructure field effect transistors (MISHFETs) up to 1000 s with a *V*_GS_ = 8 V before and after proton irradiation.

**Figure 4:**
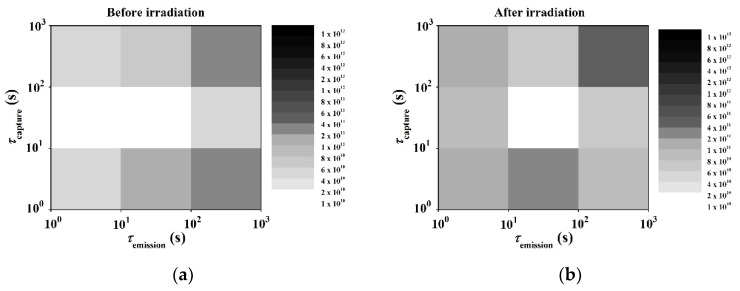
Capture emission time (CET) maps in normally-off AlGaN/GaN gate-recessed MISHFETs: (**a**) before proton irradiation and (**b**) after proton irradiation.

**Figure 5:**
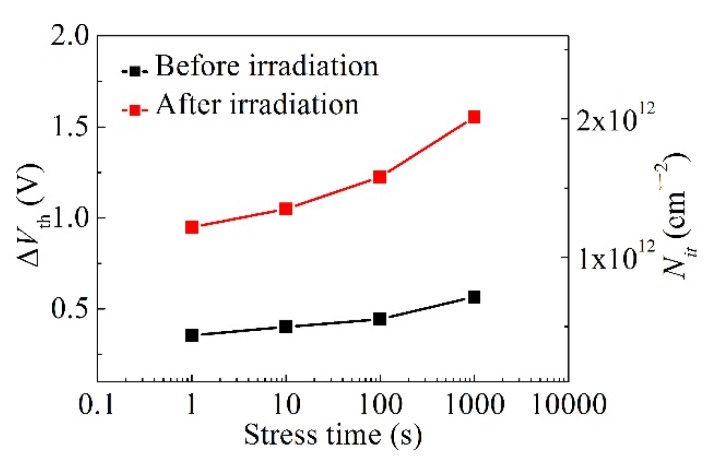
Δ*V*_th_ and $N_{it}$ drift over stress time up to 1000 s measured using a *V*_GS_ of 8 V before and after proton irradiation.

In order to investigate a broad distribution of overall traps through the gate region, which are widely believed to be applicable to GaN HFETs, capture emission time (CET) maps [21,22,23] were extracted using a stress–recovery sequence before and after proton irradiation. A CET map can be constructed from the shift of the I–V or Capacitance -voltage (C–V) characteristics. Every defect has a capture time ($\tau_{C}$) and emission time ($\tau_{e}$) during stress and recovery. Empty defects are charged and charged defects will emit its electron after the stress and recovery times of $\tau_{C}$ and $\tau_{e}$, respectively. This behavior of defects can be described using the τ-axis and is called a CET map. A typical measurement procedure of CET maps is as follows. A positive voltage is assigned to the gate to trap electrons in the interface between the insulator and GaN, and then the time-dependent recovery characteristics are observed. During the stress sequence, traps with a capture time constant smaller than the stress time are occupied. During the recovery sequence, traps with an emission time constant smaller than the recovery time are released. According to this scheme, CET maps can extract traps with a specific capture time and recovery time by repeating the time-based stress-recovery experiments. From the *V*_th_ variation value obtained through the stress–recovery experiments, the following Equation (1) is used to obtain the overall interface trap level ($N_{it}$) of the gate region:

(1) $$N_{it}=\frac{\epsilon_{0}\epsilon_{d}}{q}\frac{\Delta V_{th}}{t_{d}}$$

where $\epsilon_{0}$ and $\epsilon_{d}$ are the dielectric constants of air and insulator, respectively, and $t_{d}$ is the thickness of the insulator. $N_{it}$ was derived from *Q* = *C*·Δ*V*. The bias stress instability was induced by the 8 V of *V*_GS_. *V*_th_ was extracted at the point where the drain current of 100 μA/mm flowed into the transfer curve with *V*_DS_ = 1 V (linear region). The *V*_th_ variation through the typical stress and recovery experiments for this analysis are summarized in Figure 3. Δ*V*_th_ increased with stress time in the stress plot and decreased with recovery time in the recovery plot for several extraction points. The squares of CET maps represent the behavior of $N_{it}$ with each capture time and recovery time. CET maps obtained from the *V*_th_ variation and Equation (1) before and after proton irradiation are described in Figure 4. The overall darkening of the squares after proton irradiation qualitatively indicates the increase of trap states under the gate region. Further investigation needs to be carried out to understand the more pronounced increase in the traps within a certain time window. Figure 5 shows the change of Δ*V*_th_ and $N_{it}$ as the stress time increased up to 1000 s with a *V*_GS_ = 8 V before and after the proton irradiation. $N_{it}$ was increased from 5.6 × 10^11^ cm^−2^ to 1.6 × 10^12^ cm^−2^ at the stress time of 100 s with the *V*_GS_ of 8 V. The values of $N_{it}$ obtained from this experiment are comparable with and even lower than those in the literature with/without a gate-recessed structure [5,11,24,25,26]. Δ*V*_th_ and $N_{it}$ increased after proton irradiation. Table 1 summarizes the correlation of the interface traps states ($N_{it}$ or $D_{it}$) and proton irradiation on GaN-based transistors. $D_{it}$ has the same meaning with $N_{it}$ at the specific energy of trap levels.

**Figure 6:**
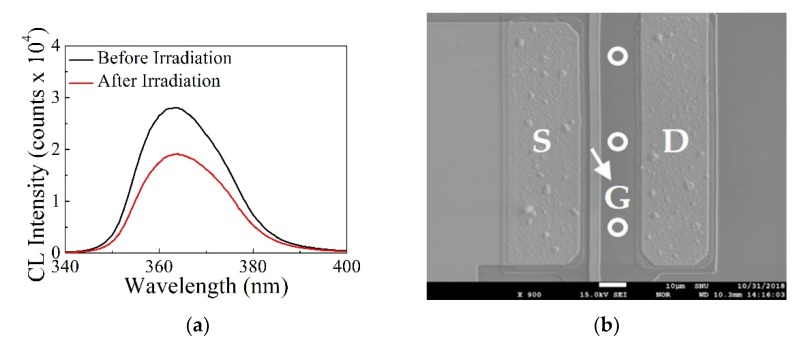
(**a**) Cathodoluminescence (CL) spectra of normally-off AlGaN/GaN gate-recessed MISHFETs before and after proton irradiation. (**b**) SEM image of the analyzed device.

The CL spectra of normally-off AlGaN/GaN gate-recessed MISHFETs shown in Figure 6a was measured to understand the proton irradiation effects on the optical properties before and after irradiation. The typical measurement procedure of CL is as follows. First, the electron beam is irradiated onto the target semiconductor. Then, the interaction of the electron beam with the target semiconductor results in the promotion of electrons from the valence band to the conduction band. When the promoted electron and hole recombine, the exposed semiconductor provides information about its optical property. This optical property can be collected using a retractable parabolic mirror. CL was measured in the access region between the gate and drain, as shown in Figure 6b. White circles indicate the points irradiated by the electron beam. The decay of CL intensity was decreased by 31.7% after the proton irradiation. This suggests that the trap states generated by the proton irradiation reduced the recombination of electron–hole pairs generated by the electron beam.

**Figure 7:**
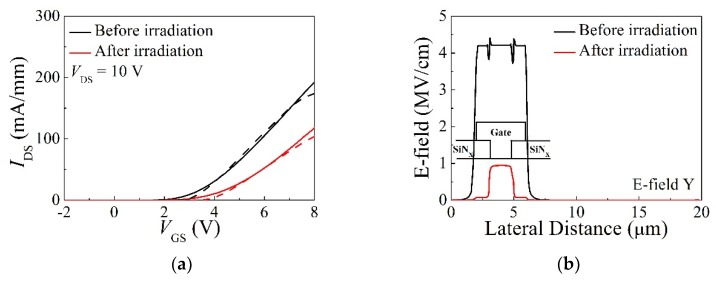
(**a**) The simulated transfer curves (dashed lines) compared with the measured ones (solid lines). (**b**) The vertical electric field distribution of the gate dielectric with *V*_GS_ = 14 V.

The main degradation mechanism of TDDB characteristics is attributed to the breakdown of the gate dielectric around the gate overhang [27]. Therefore, we carried out a TCAD simulation using Silvaco ATLAS (Silvaco Atlas, Santa Clara, CA, USA) to profile the vertical electric field distribution of the normally-off AlGaN/GaN gate-recessed MISHFETs. Figure 7 shows the simulated transfer curves compared with the measured ones (a) and the vertical electric field within the gate dielectric under the gate for *V*_GS_ = 14 V (b) before and after the proton irradiation. Proton irradiation was applied to the simulation by employing negatively charged traps in accordance with Patrick et al. [28]. Proton irradiation can generate Ga and N vacancies in the irradiated devices via collisions. Ga vacancies can act as acceptor-like traps [29], and N vacancies can act as both acceptor- and donor-like traps [30]. These two vacancies can also compensate each other, but the quantitative analysis is still unclear. However, proton irradiation results in a *V*_th_ shift in the positive direction, which can infer that the acceptor-like traps (negatively charged traps) are dominant in the irradiated device. The volume density of the traps was calculated using stopping and range of ions in matter (SRIM) and its value was reported to be about the order of 10^17^ cm^−3^ [28,31,32]. Within GaN, there are pre-existing traps with various activation energies from shallow to deep level states. Proton irradiation increases the concentration of pre-existing traps and new trap states with different activation energies [33]. Therefore, the activation energies of the trap level applied to the TCAD simulation were distributed uniformly through the bandgap of GaN. As negatively charged traps were applied, the vertical electric field of the gate dielectric under the gate was significantly decreased by 83%. CET maps and CL spectra verified the deterioration of the irradiated devices, but it was also confirmed through the TCAD simulation that the trap states induced via proton irradiation reduced the vertical electric field of the dielectric under the gate region. It is presumed that TDDB characteristics negligibly changed, even after the proton irradiation, due to the offset of these two opposite effects. C–V measurements can provide useful information for understanding the defect level and should be analyzed in our future work.

## 4. Conclusions

TDDB characteristics of normally-off AlGaN/GaN gate-recessed MISHFETs were investigated before and after proton irradiation. After proton irradiation, the irradiated devices exhibited the same *V*_GS_ dependence and a negligible change. Although the interface and trap states were deteriorated by proton irradiation, it was observed using a TCAD simulation that the vertical electric field under the gate was significantly reduced as the trap concentration increased. The field reduction via proton irradiation seemed to be linked to unchanged TDDB characteristics despite the deterioration of interface and trap states. Further investigation is needed to figure out the definite origin of the unchanged TDDB characteristics of normally-off AlGaN/GaN gate-recessed MISHFETs.

## Figures and Tables

**Table 1:** The correlation between the interface traps states and the proton irradiation on GaN-based transistors.

Operation Mode	*N_it_* or *D_it_* (cm^−2^ or cm^−2^·eV^−1^)	Irradiation Dose (cm^−2^)	Irradiation Energy (MeV)	Reference
Normally Off	1.2 × 10^12^–2 × 10^12^	5 × 10^14^	5	This work
Normally Off	1.1 × 10^12^–6 × 10^13^	5 × 10^14^	5	[5]
Normally Off	1.3 × 10^13^–2.6 × 10^13^	5 × 10^14^	5	[11]
Normally On	1.8 × 10^12^–1.8 × 10^13^	5 × 10^14^	3	[25]
Normally On	1.4 × 10^13^	10^15^	5	[26]

## References

[1] Hazdra P., Popelka S. (2017). Radiation resistance of wide-bandgap semiconductor power transistors. Phys. Status Solidi (a).

[2] Mararo J., Nicolas G., Nhut D.M., Forestier S., Rochette S., Vendier O., Langrez D., Cazaux J., Feudale M. (2010). GaN for space application: Almost ready for flight. Int. J. Microw. Wirel. Technol..

[3] Pearton S.J., Ren F., Patrick E., Law M.E., Polylakov A.Y. (2016). Review-ionizing radiation damage effects on GaN devices. ECS J. Solid State Sci. Technol..

[4] Chen J., Puzyrev Y.S., Jiang R., Zhang E.X., McCurdy M.W., Fleetwood D.M., Schrimpf R.D., Pantelides S.T., Arehart A.R., Ringel S.A. (2015). Effects of applied bias and high field stress on the radiation response of GaN/AlGaN HEMTs. IEEE Trans. Nucl. Sci..

[5] Keum D.M., Cha H.Y., Kim H. (2015). Proton bombardment effects on normally-off AlGaN/GaN-on-Si recessed MISHeterostructure FETs. IEEE Trans. Nucl. Sci..

[6] Lv L., Ma X., Zhang J., Bi Z., Liu L., Shan H., Hao Y. (2015). Proton irradiation effects on AlGaN/AlN/GaN heterojunctions. IEEE Trans. Nucl. Sci..

[7] Kim D.S., Lee J.H., Yeo S., Lee J.H. (2018). Proton irradiation effects on AlGaN/GaN HEMTs with different isolation methods. IEEE Trans. Nucl. Sci..

[8] Anderson T.J., Koehler A.D., Specht P., Weaver B.D., Greenlee J.D., Tadjer M.J., Hite J.K., Mastro M.A., Porter M., Wade M. (2015). Failure mechanisms in AlGaN/GaN HEMTs irradiated with 2MeV protons. ECS Trans..

[9] Weaver B.D., Martin P.A., Boos J.B., Cress C.D. (2012). Displacement damage effects in AlGaN/GaN high electron mobility transistors. IEEE Trans. Nucl. Sci..

[10] Lee I.H., Lee C., Choi B.K., Yun Y., Chang Y.J., Jang S.Y. (2018). Proton-induced conductivity enhancement in AlGaN/GaN HEMT devices. J. Korean. Phys. Soc..

[11] Keum D., Kim H. (2019). Proton-irradiation effects on charge trapping-related instability of normally-off AlGaN/GaN recessed MISHFETs. J. Semicond. Technol. Sci..

[12] Ambacher O., Smart J., Shealy J.R., Weimann N.G., Chu K., Murphy M., Schaff W.J., Eastman L.F.L., Dimitrov R., Wittmer L. (1999). Two-dimensional electron gases induced by spontaneous and piezoelectric polarization charges in N- and Ga-face AlGaN/GaN heterostructures. J. Appl. Phys..

[13] Smorchkova I.P., Elsass C.R., Ibbetson J.P., Vetury R., Heying B., Fini P., Haus E., DenBaars S.P., Speck J.S., Mishra U.K. (1999). Polarization-induced charge and electron mobility in AlGaN/GaN heterostructures grown by plasma-assisted molecular-beam epitaxy. J. Appl. Phys..

[14] Su M., Chen C., Rajan S. (2013). Prospects for the application of GaN power devices in hybrid electric vehicle drive systems. Semicond. Sci. Technol..

[15] Choi W., Seok O., Ryu H., Cha H.Y., Seo K.S. (2014). High-voltage and low -leakage-current gate recessed normally-off GaN MIS-HEMTs with dual gate insulator employing PEALD-SiN_X_/RF-Sputtered-HfO_2_. IEEE Electron Device Lett..

[16] Park B.R., Lee J.G., Choi W., Kim H., Seo K.S., Cha H.Y. (2013). High-quality ICPCVD SiO_2_ for normally off AlGaN/GaN-on-Si recessed MOSHFETs. IEEE Electron Device Lett..

[17] Wu T.L., Marcon D., Jaeger B.D., Hove M.V., Bakeroot B., Stoffels S., Groeseneken G., Decoutere S. Time dependent dielectric breakdown (TDDB) evaluation of PE-ALD SiN gate dielectrics on AlGaN/GaN recessed gate D-mode MIS-HEMTs and E-mode MIS-FETs. Proceedings of the International Reliability Physics Symposium (IPRS).

[18] Kim H.S., Eom S.K., Seo K.S., Kim H., Cha H.Y. (2018). Time-dependent dielectric breakdown of recessed AlGaN/GaN-on-Si MOS-HFETs with PECVD SiO_2_ gate oxide. Vacuum.

[19] Wuerfl J., Bahat-Treidel E., Brunner F., Cho E., Hilt O., Ivo P., Knauer A., Kurpas P., Lossy R., Schulz M. (2011). Reliability issues of GaN based high voltage power devices. Microelectron. Reliab..

[20] Schroder D.K. (2015). Reliability and failure analysis. Semiconductor Material and Device Characterization.

[21] Lagger P., Ostermaier C., Pobegen G., Pogany D. Towards understanding the origin of threshold voltage instability of AlGaN/GaN MISHEMTs. Proceedings of the International Electron Devices Meeting (IEDM).

[22] Reisinger H., Grasser T., Gustin W., Schluandnder C. The statistical analysis of individual defects constituting NBTI and its implications for modeling DC- and AC-stress. Proceedings of the International Reliability Physics Symposium (IPRS).

[23] Ostermaier C., Lagger P., Reiner M., Pogany D. (2018). Review of bias-temperature instabilities at the III-N/dielectric interface. Microelectron. Reliab..

[24] Wang Y., Wang M., Xie B., Wen C.P., Wang J., Hao Y., Wu W., Chen K.J., Shen B. (2013). High-Performance normally-off Al_2_O_3_/GaN MOSFET using a wet etching-based gate recess technique. IEEE Electron Device Lett..

[25] Zheng X.F., Dong S.S., Ji P., Wang C., He Y.L., Lv L., Ma X.H., Hao Y. (2018). Characterization of bulk traps and interface states in AlGaN/GaN heterostructure under proton irradiation. Appl. Phys. Lett..

[26] Kim B.J., Ahn S., Ren F., Pearton S.J., Yang G., Kim J. (2016). Effects of proton irradiation and thermal annealing on off-state step-stressed AlGaN/GaN high electron mobility transistors. J. Vac. Sci. Technol. B.

[27] Alamo J.A., Guo A., Warnock S. (2017). Gate dielectric reliability and instability in GaN metal-insulator-semiconductor high-electron-mobility transistors for power electronics. J. Mater. Res..

[28] Patrick E., Law M.E., Liu L., Cuervo C.V., Xi Y., Ren F., Pearton S.J. (2013). Modeling proton irradiation in AlGaN/GaN HEMTs: Understanding the increase of critical voltage. IEEE Trans. Nucl. Sci..

[29] Look D.C. (2001). Defect-related donors, acceptors, and traps in GaN. Phys. Status Solidi B.

[30] Ganchenkova M.G., Nieminen R.M. (2006). Nitrogen vacancies as major point defects in gallium nitride. Phys. Rev. Lett..

[31] Ziegler J.F., Ziegler M.D., Biersack J.P. (2010). SRIM—the stopping and range of ions and matter (2010). Nucl. Instrum. Methods Phys. Res. B.

[32] Lv L., Ma J.G., Cao Y.R., Zhang J.C., Zhang W., Li L., Xu S.R., Ma X.H., Ren X.T., Hao Y. (2011). Study of proton irradiation effects on AlGaN/GaN high electron mobility transistors. Microelectron. Reliab..

[33] Zhang Z., Arehart A.R., Cinkilic E., Chen J., Zhang E.X., Fleetwood D.M., Schrimpf R.D., McSkimming B., Speck J.S., Ringel S.A. (2013). Impact of proton irradiation on deep level states in n-GaN. Appl. Phys. Lett..

